# Age-Associated Perceptions of Physical Activity Facilitators and Barriers Among Women in Rural Southernmost Illinois

**DOI:** 10.5888/pcd13.160247

**Published:** 2016-09-29

**Authors:** Kristine Zimmermann, Leslie R. Carnahan, Nadine R. Peacock

**Affiliations:** Author Affiliations: Leslie R. Carnahan, Nadine R. Peacock, University of Illinois at Chicago, Chicago, Illinois.

## Abstract

**Introduction:**

Women living in rural areas in the United States experience disproportionately high rates of diseases such as obesity and heart disease and are less likely than women living in urban areas to meet daily physical activity (PA) recommendations. The purpose of our research was to understand age-specific perceptions of barriers and facilitators to rural women engaging in PA and to identify strategies to promote PA among these women.

**Methods:**

As part of a community health assessment to learn about women’s health issues, 110 adult women participated in 14 focus groups. The women were divided into 4 age groups, and focus groups were held in various community settings. We used qualitative analysis methods to explore themes in the women’s narratives, including themes related to PA knowledge, PA behavior, and access to PA facilities.

**Results:**

Participants described multiple and often conflicting individual, social, and environmental barriers and facilitators to PA. Several barriers and facilitators were shared across age groups (eg, competing priorities and inadequate knowledge about PA’s role in disease prevention and disease management). Other barriers (eg, illness and injury) and facilitators (eg, PA as a social opportunity) differed by age group.

**Conclusion:**

Rural women in southernmost Illinois have often contradictory barriers and facilitators to PA, and those barriers and facilitators are different at different points in a woman’s life. Our findings suggest the need for multilevel, multisector approaches to promote PA. Additionally, this research supports the need for tailored PA promotion programs for rural women to address the barriers these women face across their lifespan.

## Introduction

Insufficient physical activity (PA) is associated with a high risk for health conditions such as obesity, cardiovascular disease, and risk factors for cardiovascular disease (1,2). Lack of PA also contributes substantially to health care costs in the United States (3). Residents of the rural United States are less likely than their urban peers to meet PA recommendations and are more likely to be physically inactive (4). Overcoming the obesity and chronic disease disparities facing rural populations (5,6) requires increasing the number of people who engage in PA in rural communities (4).

The level of PA by rural adults is associated with individual, social, and environmental factors such as self-efficacy, family and peer support, and access to parks and exercise facilities (7,8). Rural women also report lack of time, motivation, or energy as barriers to PA (9). However, these qualitative studies (7–9) did not consider rural women’s life stages when examining the women’s perceptions about barriers and facilitators to PA. Using a life course perspective when examining rural women’s level of PA allows us to place women’s barriers to and motivators for PA in the context of a time and place. The results of such an examination can, therefore, contribute to 1) a better understanding of how and why rural women engage or do not engage in PA and 2) improved interventions to increase PA by rural women (10). Tailoring interventions to each of women’s life stages can be beneficial for motivating women to begin and maintain PA habits (11).

In the rural 7 southernmost (S7) counties of Illinois, more than two-thirds of women are overweight or obese, more than half fail to meet recommended PA guidelines, and 14% are inactive (12). We present the findings from a focus group study with S7 women. Our objective was 1) to learn the perceptions about facilitators and barriers to PA of rural adult women at various ages and 2) to recommend ways to improve public health interventions to increase PA by women living in rural areas.

## Methods

The S7 counties of Illinois are Alexander, Hardin, Johnson, Massac, Pope, Pulaski, and Union. The 2010 population in the region was 69,008, and the area is more than 2,000 square miles (13). According to the 2013 Rural-Urban Continuum Codes, which are used to classify counties as metropolitan or non-metropolitan by degree of urbanization based on population size and proximity to an urban area, 6 of the S7 counties were classified as nonmetropolitan (14). The S7 population is primarily white; in 2010, however, 10.5% of residents were African American, and 2.8% were Hispanic (13).

In 2011, the Southern Seven Coalition for Women’s Health (SSCWH), which has a mission to promote health and wellness for women and their communities in the S7 region, conducted a comprehensive assessment of women’s health in the region (15). The assessment included 14 focus groups with community women from across the lifespan. SSCWH researchers conducted at least 2 focus groups in each county, and at least 3 focus groups for each of 4 age groups: 18 to 30 years, 31 to 50 years, 51 to 70 years, and older than 70 years. Because of their small population size, Pope and Hardin counties were combined into a single recruitment area. Focus groups took place during February and March 2011. The University of Illinois at Chicago Institutional Review Board approved the research.

To recruit a diverse sample of women by age, race/ethnicity, county of residence, and socioeconomic status, the study was advertised in newspapers, flyers in the community, and announcements in church and community newsletters. Interested women signed up for focus groups through the local health department, and health department personnel screened women for eligibility on the basis of age and county of residence. Focus groups were held in public locations, such as clinics, churches, hospitals, a public library, and senior living facilities.

The SSCWH’s academic partner (University of Illinois at Chicago, Center for Research on Women and Gender) trained health educators from the local health department to facilitate the focus groups, and the facilitators obtained informed consent from participants before conducting the focus groups. Using a semi-structured guide, facilitators elicited perceptions about women’s health needs and community needs and assets. Using probes, the facilitators encouraged natural conversation among the participants but guided the discussion to ensure all topics in the focus group guide were covered. Focus groups lasted on average a little more than an hour and ranged from 45 to 90 minutes. Each participant received a $15 gift card as an incentive to participate. Focus groups were audio-recorded and transcribed verbatim.

The goals of this secondary analysis were to understand rural women’s perspectives about personal and community health, to advance public health knowledge about rural women’s health, and to understand rural women’s engagement in and access to health promoting activities. We used an inductive process to explore patterns and themes in the data; however, our analysis approach was also influenced by the original goals of the focus groups. Specifically, we sought to understand the factors (barriers and facilitators) that affect the health of S7 women and their communities from the perspective of women of different age groups. We used ATLAS.ti, version 7 (Scientific Software Development GmbH) for the analysis.

Two members of the research team (L.C., K.Z.) reviewed the focus group transcripts and took notes to record their thoughts as they read the transcripts and to track their observations about prominent patterns. We used these notes to develop the initial codebook. For the final codebook, we used an iterative process to code transcripts individually, after which all team members met to discuss coding discrepancies. Our codebook had 45 codes, separated into 8 broad categories: access, demographics, environment, food, health, heath care, illness or disease, and PA.

Using the final codebook, we calculated Cohen’s κ and crude agreement on a set of independently segmented and coded transcripts multiple times until we reached an acceptable agreement score (mean κ = 0.89; crude agreement = 98.1%). After reaching agreement, 1 team member (L.C.) coded the remaining transcripts, and another team member (K.Z.) reviewed the coded transcripts.

We explored prominent patterns using “queries” and other Atlas.ti analysis tools. We met regularly to discuss our summary findings and conducted additional queries to confirm themes. We also met frequently with the senior qualitative researcher (N.P.) to discuss our analysis approach and findings.

## Results

A total of 110 women, of whom 31 were African American and 79 were white, participated in the 14 focus groups across the 7-county area (Table 1). Among the prominent themes from the focus groups with regard to PA was that women can experience multiple, often contradictory barriers and facilitators at the individual, social, and physical environment levels, which affect their participation in PA. In addition, these facilitators (Table 2) and barriers (Table 3) may differ depending on a woman’s age.

**Table 1:** Characteristics of Focus Group Participants (N = 110), Southern Seven Region of Illinois, 2011

Characteristic	n (%)
**Race/Ethnicity**	
Non-Hispanic black	31 (28)
Non-Hispanic white	77 (70)
Hispanic white	2 (2)
**Age, y**	
18–30	26 (24)
31–50	24 (22)
51–70	27 (25)
>70	33 (30)
**County of residence**	
Alexander	27 (25)
Johnson	9 (8)
Massac	9 (8)
Pope or Hardin	31 (28)
Pulaski	18 (16)
Union	16 (15)

**Table 2:** Sample Quotations Describing Facilitators to Engaging in Physical Activity by Rural Women in the Southern Seven Region of Illinois, 2011

Theme	Quotations
**Individual Facilitators**	
**Knowledge about or experiences with the benefits of physical activity**	**Pulaski County, 18–30 age group:** I just do what my mamma do when she’s stressed out. She just has to go for a walk, and you be surprised how far you can walk and walk back, and by that time you be calm and you wanna go sit down anyway.
	**Pope or Hardin County, 31–50 age group:** [Name] and I weigh every morning, and I have high cholesterol, so we try to keep our weight down. . . . I think [exercise] might be, is what we need to do to try to stay healthier.
	**Alexander County, 51–70 age group:** I was walking to town to lower my cholesterol. It was like three or four hundred every time I’d go to the doctor . . . and it’s 137. It’s excellent now. I want to live.
	**Alexander County, older-than-70 age group:** Particularly in me and my sister's family, there's a high rate of cancer and high blood pressure and diabetes and glaucoma. You name it, we got it. . . . Since I’ve retired I started walking. I used to hate walking, I like it now. I even walked in the rain this morning, on the balcony, back and forth.
**Social Environment Facilitators**	
**Desire to be a role model for or spend time with one’s children**	**Pope or Hardin County, 18–30 age group**: Participant 1: I try to set an example for my 6-year-old. Being overweight kind of runs in our family, and she doesn’t have the best eating habits. So, like I try to help her eat, you know, better. And then exercising, we’re really into exercising two or three days a week, you know. I make sure to do something so she can look up to me. So maybe help her, hopefully her health will not be as bad as some of my family. Participant 2: Yeah, I’m with [name]. I mean, you want to take care of your own health, but then when you have kids you want to be there to help support them and make sure they're brought up right and know what’s healthy for them and set a good example.
	**Union County, 31–50 age group:** [I] just wanna set a good example for my kids, so that’s kinda where I’m at right now. . . . So, if I can get my life and my choices and my exercise, then maybe that could set a good example for them.
**Desire to do things with and for family**	**Massac County, 51–70 age group:** Well, a lot of other people have children, or grandchildren, or, you know, people that always a bit dependent on ’em. And you want to stay healthy . . . to be able to do things for them also.
	**Alexander County, older-than-70 age group:** I like feeling good. And I like looking good. And I like being around my grandkids and doing things for them, and my kids, too.
**Social support**	**Johnson County, 51–70 age group:** But I do teach water aerobics down here at the [name of business]. . . . I tell the gals we got up, we got dressed, we came to town, that’s more than you probably would have done sitting around the house in your jammies all day long. . . . I think the social part of our gathering is as important as the actual movement.
	**Massac County, 51–70 age group:** I think, by going to the pool, there is a set of friends slash acquaintances there. And we are all concerned about each others’ families and who’s sick and who has had surgery and all that. Then there’s the group at Curves.
**Women motivate one another**	**Moderator**: Anything else that you can think of that we can gear specifically toward women? **Pulaski County, 18–30 age group:** Like a health [class] that deals with everything like, maybe one week you focus on physical exercise, a different week you can focus on like eating habits, and then just go on from there. . . . I think it’s better if it’s like, you know, women because they just motivate each other to keep going.
	**Union County, 31–50 age group:** I think when you’re talking about women in particular, programs put on by women for women, I think they really, really kind of hit home.
	**Johnson County, 51–70 age group:** I probably got approximately 15 people walking. You should see the people that walk in the campground. . . . Most people have golf carts and that, but . . . they’ll walk, and it’s just amazing.
	**Moderator**: Are there any specific activities or services that could be targeted just to women? **Alexander County, older-than-70 age group:** Participant 1: Maybe an exercise group where they got together. Different forms of exercise such as maybe the gym exercise machines or swimming. Some people can do swimming. Participant 2: Pair up and walk. I don't like to walk by myself but I do like to walk.
**Physical Environment Facilitators**	
**Parks and trails available to residents**	**Johnson County, 18–30 age group** As my kids get older, and are done with the playground, they ask if we can just go walk a trail, and we can do different ones each time and we can veer off a little bit . . . and it’s within driving distance so you can afford to go.
	**Union County, 31–50 age group:** We have Giant City State Park, and then we have walking trails and different places. We have a lot of great beauty in southern Illinois and I think that’s nice. We can go to the Garden of the Golds and there’s a lot of different places. It doesn’t even have to cost any money, which is a huge thing.

**Table 3:** Sample Quotations Describing Barriers to Physical Activity by Rural Women Living in the Southern Seven Region of Illinois, 2011

Theme	Quotations
**Individual-Level Barriers**	
**Lack of awareness about the importance of physical activity or how to be physically active**	**Pulaski County, 18–30 age group:** One of my neighbors, since she’s a diabetic and all, I usually have to help her out . . . my dad said that sometimes she does have to lift up her arms so she can get a little bit of exercise a little bit at a time, then stop, then do it again. I keep on telling her that but she just won’t listen to me.
	**Pope or Hardin County, 31–50 age group:** I think it goes back to the exercise facilities . . . we need one of those. And the education, it goes right back to that, we need more of that.
	**Alexander County, 51–70 age group:** I think we need somebody just to show us, you know, how to exercise and stuff.
	**Union County, older-than-70 age group:** . . . probably education classes, is probably what it needs to be, but then you gotta have people go to the education classes.
**Personal health challenges**	**Alexander County, 31–50 age group:** Participant 1: Lack of exercise. Participant 2: ’Cause you’re depressed and you don’t feel like getting up and doing anything. Stress.
	**Massac County, 51–70 age group:** I don’t walk for exercise. At my job I do a lot of walking. But it’s just . . . I don’t have the stamina. I don’t have the energy. Now I’m starting to hurt when I get out of bed.
	**Johnson County, 51–70 age group:** I have rheumatoid arthritis, so I do definitely have some challenges as well. . . . I have tried my challenge at Zumba, and I love it, but I think it has done me a disservice . . . since going, I have started really hurting again.
	**Alexander County, older-than-70 age group:** I used to like to bike ride before I had my back injury, and it was good exercise. And I stayed thinner.
**Difficult to maintain physical activity routine**	**Alexander County, 51–70 age group:** And I need to improve my health to the best that I can. I have some health issues, too . . . I need to get to, and maintain, a healthy weight, and I need to develop a regular exercise program . . . I need the motivation.
	**Pope or Hardin County, older-than-70 age group:** I walked all the time, and I did everything I was supposed to do, and I lost a lot of weight and got good and healthy. Well, I fell off that wagon.
**Social Environment Barriers**	
**Competing priorities, caretaking responsibilities, lack of time**	**Moderator**: Anything else that you think can be done to prevent these things; the heart disease, obesity, diabetes, or cancer? **Johnson County, 18–30 age group:** Participant 1: Like exercise classes, I know they were having like Zumba ones at the high school now, a lot of people are really into that. . . . Participant 2: I’ve got kids so I stick with walking through town when they’re in school.
	**Union County, 31–50 age group:** We’re so busy being mothers, daughters, sisters, aunts, cousins, and we wanna take care of everyone and we neglect ourselves a little bit, I think.
	**Pulaski County, 51–70 age group:** If you work then you come to exercise class, then you gotta go home and cook supper, then you gotta clean, you gotta do the laundry. And, you know, that’s one thing that women that work do not have time for. They don’t have time to stop what you’re doing, and travel somewhere. . . . Women don’t have time enough to do all of their normal stuff of the day, and fit in exercising time for themselves.
	**Union County, older-than-70 age group:** I kinda got into quite a bit of volunteering and [my exercising] dropped off, and never got started back.
**Sedentary society**	**Johnson County, 18–30 age group:** I believe we are becoming more of a sedentary society and I believe that exercise is the key to health, and how do you promote that to people and keep people involved in that?
	**Union County, 31–50 age group:** There’s not the regular, “You need to go outside and play” kind of attitude. It’s just an expectation, you know, that it’s okay to be sedentary.
**Physical Environment Barriers**	
**Natural environment factors**	**Pulaski County, 18–30 age group:** Participant 1: I think people in my community need to exercise more. . . . You don’t, like, see a lot of people outside. You see a lot of them indoors . . . they usually in the house, watching TV, on the computer, not doing anything active. **Moderator**: Okay, you think it’s because they’re not sure what to do or is there another reason maybe why? Participant 1: It’s too hot. Participant 2: The mosquitoes down here are really big. So it’s like you can either stand out in the sun and get sunburnt or you come out at night and get ate up by mosquitos, so you just choose to be in the house ’cause you don’t want to put on all that bug repellant and then you can’t get it all off.
	**Pope or Hardin County, 31–50 age group:** I love the outdoors. I’d love to be able to ride a bike around. But these hills? I don’t think so. I’m from the city, so it’s all flat where I’m from.
	**Johnson County, 51–70 age group:** And with arthritis coming on and everything, I find that if I keep walking, and in the wintertime it’s very hard to do that, so I have to do exercises.
**Lack of access to local, affordable, appropriate physical activity resources**	**Massac County, 18–30 age group:** You have to pay a ton to be healthy. To get into a gym now you gotta pay a lot. . . . the poor community cannot, in the wintertime, especially, get out and be active. It’s cold. It’s not healthy on you to do that.
	**Pope or Hardin County, 31–50 age group:** We don't have anything locally or close by that I know of that we can consistently go and exercise, if you’re on a weight program . . . I mean I think that there’s a church that does Zumba once a week or something like that, but that’s it as far as I know.
	**Alexander County, 51–70 age group:** They need a community center here for people to be able to exercise. And I’m not talking about set-up like they got here, but it seems like it’s more geared toward men instead of women. I mean, women probably do wanna do weight lifting, but not with them old heavy [men].
	**Pulaski County, 51–70 age group:** Participant 1: In Grand Chain we have Zumba class at the old high school, but it’s only twice a week and it’s 5:30 to 6:30 . . . And now at the college, I know they have that . . . Participant 2: They do have it five nights a week there. They’re at different times. . . . Participant 3: Now we have to drive. . . . Participant 4: You take 20 or 30 minutes out of your day. Well wait, you know, that takes an hour. Now, it’s 2 hours out of your day that you already spent 8 doing something somewhere else.
	**Pope or Hardin County, older-than-70 age group:** Participant 1: The [physical therapy center] has an exercise place, you can go exercise. . . . **Moderator**: So there is someplace to go in the winter months when it’s too cold, or too hot in the summertime? Okay. Participant 2: . . . They cut their hours . . . they don’t have enough business . . . but you can, if you’re a senior citizen, you can use their equipment, when they are open, for $15 a month, and I think that’s very reasonable.

### Individual factors

Comments from participants of all age groups reflected their level of awareness of the personal benefits of PA on their own physical and mental health and on their motivation to be active (Table 2). Participants reported engaging in PA to relieve chronic pain and manage illness; to improve mental health, including to reduce stress; and to feel more energetic. Participants also described wanting to avoid the health conditions that their family members experienced, such as heart disease, cancer, or premature death. One participant reported that a motivation to be active, particularly among older women, was enjoyment of PA and the desire to live long and healthy lives.

The consensus across age groups was that lack of awareness about the importance of PA or how to be active was a barrier to engaging in PA (Table 3). In addition, all participants, except those aged 18 to 30, discussed physical and mental health challenges (including illness, injury, and lack of energy) that interfered with their ability to be active. Participants in the 51-to-70 and older-than-70 groups also indicated that maintaining a PA routine was often a challenge because of difficulty staying motivated to exercise and not making PA a priority.

### Factors related to the social environment

Participants in all age groups noted as barriers to PA participation both time constraints and competing priorities, including employment, childcare, and household responsibilities (Table 3). These factors were reported to interfere with women’s ability to engage in a variety of self-care activities, including PA. In particular, women’s caretaking role may lead them to focus on others instead of themselves. Women in the age groups 18-to-30 and 31-to-50 described their communities as sedentary, with inactivity being the norm.

Participants drew connections between individual factors and social roles in describing their motivations to be active. They described the importance of PA in allowing them to be healthy and, therefore, allowing them to better care for loved ones (Table 2). Young women described engaging in PA as a way to be a role model for children and other family members, and they discussed PA as something for families to do together. Older participants expressed their desire to stay healthy to care for and enjoy their grandchildren, and that being physically active was a way to maintain their health. Women aged 51 to 70 also discussed PA as a social opportunity, where the social support received through exercising together was an important aspect of the experience. In all age groups, participants talked about women’s roles as motivators for other women.

### Factors related to the physical environment

Participants’ descriptions of the role of both the natural and built environment in supporting or inhibiting PA varied across groups. Young women noted the attractive natural environment of the region, and discussed the availability of parks and trails in the region as good options for themselves and their children to engage in PA (Table 2). However, participants in all age groups, except in the older-than-70 group, described barriers associated with the natural environment: weather conditions, mosquitoes, and topography, which they saw as barriers to engaging in outdoor PA (Table 3).

Similarly, some participants described options for PA as accessible gymnasiums and community PA classes; however, most participants perceived PA options largely as unavailable, unaffordable, or inaccessible because of hours of operation, cost, or distance from home (Table 3).

## Discussion

This study examined the age-associated barriers and facilitators to rural women engaging in PA. Similar to other research findings, our findings suggest that, although rural women may understand the importance of being physically active, knowledge alone is insufficient for motivating them to be active (16). Rather, an interaction among personal, social, and environmental factors either support or prevent rural women’s engagement in regular PA. In addition, personal, social, and environmental factors affect women differently depending on their life stage: supportive and preventive factors change over a woman’s lifespan. Furthermore, changes over time in women’s roles in society require not only that intervention programs have age-specific strategies to promote PA, but dynamic strategies that appeal to different generations of women (10).

Our findings are also consistent with other research findings with regard to social factors: social support, competing priorities, and social norms influence whether rural women participate in PA (17–19). By analyzing responses by age, we learned that young women did not describe exercise as an activity to do with other women. Young women are likely to have work, household, and family commitments that compete with social activities, including PA. For these women, their time and ability to travel to PA classes can be limited, so that PA may be more practical as a solo or family activity or as an activity incorporated into their daily routine. Conversely, when women are older, they may engage in PA as a way to spend time with other women (eg, through PA classes or walking groups).

We found, as did other researchers, that affordability for and accessibility to PA resources in rural areas are important factors in whether women in those areas engage in PA (8,17). However, we learned that perceptions about and experiences with the rural physical environment also differ by age. Young women are more interested than older women in free spaces for outdoor recreational activity, spaces where they can be active with their children. Young women may not have time to attend structured classes because of cost, scheduling, or other commitments. Few women older than 50 in our focus groups discussed outdoor PA resources, which may be because of concerns about safety related to walking outdoors in a rural community. Furthermore, the older women may have more flexible schedules that allow them to participate in structured PA groups and classes, and they may have access to more affordable PA options because of senior discounts.

The differences and fluidity in the barriers and facilitators that rural women face across the lifespan highlight the importance of establishing and reinforcing PA patterns among children and young adults to help foster lifelong PA habits. In addition, our research suggests that the goal of having rural women engage in PA requires tailored programs and outreach to women who are at different points in their lifespan. Tailored programs should consider the age of the women targeted, their health status, and their access to PA resources in their community (20). This lifespan approach should have multiple benefits, including reducing obesity and cardiovascular disease and improving cardiorespiratory fitness (21). Possible strategies include working with health care providers and health systems to promote PA in health care settings, and tailoring PA promotions to the targeted women’s PA levels, age, ability, health history, and health status (21–23). Rural health systems could also offer group PA programs for adults older than 50.

Consistent with ecological models of health (24), this research supports using multilevel strategies to promote PA to increase women’s knowledge and awareness of the benefits of PA and to decrease barriers to their engaging in PA. Examples of such strategies include structured worksite PA programs (18); community indoor recreational spaces developed in collaboration with schools and churches to overcome difficulties with accessibility to PA associated with cost, travel distance, time, safety, and other factors (19,25); and interventions to raise awareness and foster social support for PA (26). Using technology such as computers or smart phones to educate and support women to engage in PA at home (23,27) is another strategy to consider.

To counteract the persistent barriers to PA that rural women face, ongoing support that encourages them to engage in PA is needed. Specifically, focus group participants described such barriers as personal illnesses and injuries and caretaking a sick parent or spouse. PA interventions for rural women should target them during significant time points, including after a chronic disease diagnosis, or after the disease diagnosis of a parent, family member, or partner (28). Interventions may also be relevant during other significant times, such as during pregnancy and after childbirth (29,30).

Our research findings have limitations in that the focus groups were conducted to examine broad women’s health needs in the community and were not specifically focused on PA. Further qualitative research is needed to understand each thematic area better. Narrower age groups would also ascertain more specifically the age-specific barriers and facilitators to rural women engaging in PA. In addition, the generalizability of our findings are limited for several reasons. Those who participated in the focus groups were self-selected and may be more attuned to health issues than women in the general population. Because of the heterogeneity of rural communities across the country, the generalizability of this research to other US rural populations may be limited.

Our research is unique in that it explored age-associated barriers and facilitators to engaging in PA for rural women in southernmost Illinois. Reducing obesity and obesity-associated diseases among rural women requires promoting positive habits throughout the lifespan. Such promotions must be done both by providing opportunities for PA and education about the benefits of PA and by creating an environment that supports PA. By understanding the complex interaction of PA barriers and facilitators at multiple levels, we can develop and test interventions that reduce these barriers and capitalize on what motivates women at different times in their lives.
